# Genome-Wide Analysis of Invertase Gene Family, and Expression Profiling under Abiotic Stress Conditions in Potato

**DOI:** 10.3390/biology11040539

**Published:** 2022-03-31

**Authors:** Asad Abbas, Adnan Noor Shah, Anis Ali Shah, Muhammad Azhar Nadeem, Ahmad Alsaleh, Talha Javed, Saqer S. Alotaibi, Nader R. Abdelsalam

**Affiliations:** 1 School of Horticulture, Anhui Agricultural University, Hefei 230036, China; asadabbas20088@yahoo.com; 2 Department of Agricultural Engineering, Khwaja Fareed University of Engineering and Information Technology, Rahim Yar Khan 64200, Pakistan; 3 Department of Botany, University of Education Lahore, Lahore 54770, Pakistan; anisalibot@gmail.com; 4 Faculty of Agricultural Sciences and Technologies, Sivas University of Science and Technology, Sivas 58140, Turkey; manadeem@sivas.edu.tr; 5 Molecular Genetic Laboratory, Science and Technology Application and Research Center, Institute for Hemp Research, Yozgat Bozok University, Yozgat 66200, Turkey; ahmad.alsaleh@bozok.edu.tr; 6 Department of Agronomy, University of Agriculture Faisalabad, Faisalabad 38040, Pakistan; mtahaj@fafu.edu.cn; 7 College of Agriculture, Fujian Agriculture and Forestry University, Fuzhou 350002, China; 8 Department of Biotechnology, College of Science Taif University, P.O. Box 11099, Taif 21944, Saudi Arabia; saqer@tu.edu.sa; 9 Agricultural Botany Department, Faculty of Agriculture (Saba Basha), Alexandria University, Alexandria 21531, Egypt; nader.wheat@alexu.edu.eg

**Keywords:** *Solanum tuberosum*, invertase genes, carbon metabolism, abiotic stress, qRT-PCR, gene expression

## Abstract

**Simple Summary:**

Invertase genes are among the important genes responsible for carbon metabolism in plants, significantly contributing to plant development and stress responses. In this study, for the first time, we performed genome-wide analysis for Invertase gene family in potato, identified and conducted expression profiling in different tissues by RNA seq analysis and validated it by Q-PCR. We also performed invertase family genes expression profiling under drought, salt and heat stress to elucidate their involvement in stress responses. Findings of this study will be helpful for future functional and genetic studies not only in potato but also in other plants.

**Abstract:**

The potato is one of the most important and valuable crops in terms of consumption worldwide. However, abiotic stressors are the critical delimiters for the growth and productivity of potato. Invertase genes play key roles in carbon metabolism, plant development, and responses to stress stimuli. Therefore, a comprehensive genome-wide identification, characterization and expression analysis of invertase genes was performed in the potato. The current study identified 19 invertase genes, randomly distributed throughout the potato genome. To further elucidate their evolutionary, functional and structural relationship within family and with other plant species, we performed sequence and phylogenetic analysis, which segregated invertase genes into two main groups based on their sequence homology. A total of 11 genes are included in acidic invertases and 8 genes are in neutral or alkaline invertases, elucidating their functional divergence. Tissue specific expression analyses (RNA sequencing and qRT-PCR) of different plant tissues showed differential expression pattern. Invertase genes have higher expression in flower, leaf, root and shoot tissues, while under abiotic stress conditions, the expression of the invertase gene is significantly upregulated. Results of this study revealed that vacuolar and cell wall destined invertases are mainly the functional member genes of the invertase family. This study provides comprehensive data and knowledge about *StINV* genes in *Solanum tuberosum* for future genetic and epigenetic studies.

## 1. Introduction

Higher plants are known for their carbon autotrophy; sucrose and its cleavage products glucose and fructose are the main participants of carbohydrate displacement in higher plants [1,2]. Invertases are termed as omnipresent enzymes (glycoproteins) which sunder sucrose into glucose and fructose. Plant invertases have a crucial role in carbon partitioning from origin tissues (autotrophic leaves) to the storage tissues, such as seeds, tubers and fruits; furthermore, invertases are involved in the plant development and responses to biotic and abiotic stresses [3,4]. Depending upon solubility, pH and origin invertases can be divided into three isoenzyme types. Type one is soluble neutral/alkaline invertases that are present in the cytoplasmic region; the second is cell wall destined acidic invertases that are responsible for the sucrose conversion in the apoplastic region also known as apoplastic invertases; the third and last is vacuolar invertases that are soluble acidic invertases restricted to the vacuole [5,6].

Cell wall and vacuolar invertases are also known as β-fructofuranosidases; their enzymatic and biochemical properties are similar, and they show identity in sequence and have few conserved motifs (WECP(V)D, RDP and NDPNG(A)) [1,7]. Motif WECP(V)D incorporates Val residue in vacuolar invertases, and in cell wall invertases, the Val residue is substituted by Pro residue. In *Chenopodium rubrum*, the presence of WECPD in cell wall invertases renders its higher pH and raffinose degradation rate in comparison to the presence of WECVD in vacuolar invertases [8]. Cell wall invertases hydrolyzes sucrose, then the uptake of reduced sugars is catalyzed by the STPs (sugar transport proteins). STPs have the ability to sense sugars, most of their expression is in the vegetative parts and the developing seeds. They are supposed to have many physiological functions which equip the plants to utilize sugars directly in growth or in other physiological mechanisms [9], whereas vacuolar invertases are responsible for the accumulation of reducing sugars, noticed during cold storage (cold induced sweetening) causing acrylamide production in potato products, considerably reducing their quality [10,11]. Vacuolar and cell wall invertases have the best catalytic activity within the pH range of 4 to 4.5 [3]. Very little is known about the physiological effects of the neutral/alkaline invertases due to their low enzymatic activity, as they do not belong to the glycosylates and fructofuranosidases and solely degrade sucrose [1].

Acidic invertases are much more divergent than neutral/alkaline invertases; as in sugarcane and sorghum, gene duplication was observed mainly in the acidic invertases. The presence of the invertase gene family claims the link of evolution of algae and higher plants through the endo symbiotic event in which endosymbiont (cyanobacteria) invaded the respiratory eukaryote, which is non-photosynthetic [12]. Plant acid invertases have similarity with the invertases of respiratory eukaryotes (yeast, aerobic bacteria) and plant neutral/alkaline invertases have similarity with cyanobacteria invertases [13,14].

The potato is the most produced ‘non-cereal’ crop in the world. It is a rich source of carbohydrate, fiber vitamins and other dietary nutrients, and hence the potato significantly contributes to food security. However, the changing environment heat, salt and drought stresses are causing significant reduction in crop production [15]. In the recent study, we simulated heat, drought, and salt stresses to check the activity of invertase genes. 

In spite of their extensive involvement in the plant metabolism, a lot of research was done on invertase genes, but still the invertase gene family was explored in only a handful of studies and thus, not much is known about it in major crops, such as potato, especially under abiotic stress conditions. Biotic and abiotic stresses accompany oxidative stress. Invertases help in reducing oxidative damage by managing ROS reactive oxygen species in mitochondria and balance between ADT and ATPs. In the potato, invertases are involved in CIS (cold-induced sweetening), as during cold storage, invertases have a determinant effect on the tuber sugar content [16]. Mainly, carbon is stored as starch in the tuber. When stored for extended periods of time under 4 °C, the conversion of starch to sugars was noticed as an adaptive response to cold stress [17]. Higher contents of reducing sugars and amino acids results in a non-enzymatic Maillard reaction during deep frying at high temperature, deteriorating the potato products quality with elevated concentrations of acrylamide, which is carcinogenic and hazardous for human health [18]. In previous studies, cold stress was focused and invertase genes activity in other abiotic stress conditions was neglected. In this study, for the first time, we have explored *StINV* activity under heat, drought and salt stresses. The invertase gene family was not studied before in the potato as a whole. The primary aim of this study was also to identify and annotate invertase family genes and to analyze tissue specific expression, which will be helpful for future epigenetic studies.

## 2. Materials and Methods

### 2.1. Database Search and Sequence Retrieval

Firstly, invertase gene sequences were identified in *Arabidopsis thaliana* using the database TIAR10 [19]. The retrieved *Arabidopsis* genomic sequences were translated using ExPASy translate tool (expert protein analysis system, https://web.expasy.org/translate/, accessed on 7 August 2021) to obtain the protein sequences of invertase gene family participant genes. To discover all the member genes of the invertase gene family in *Solanum tuberosum*, functionally annotated known protein sequences of *Arabidopsis* were blast searched in database (spud DB) using JGI (Joint Genome Institute) Phytozome. JGI (Joint Genome Institute) provided us with genomic, transcript, CDS, peptide sequences along with gene annotation, functional domains, and description about the physical, chemical properties of the genes [20]. In the case of genes with more than one primary transcript being considered for analysis, all the superfluous and incomplete sequences were manually excluded from analysis. 

### 2.2. Phylogenetic Analysis and Gene Structure Illustration 

Using Phytozome version 12.1 genomic, transcript, CDS and protein sequences were downloaded. For phylogenetic analysis, multiple alignment of the full-length protein sequences was done by using ClustalX2, then the unrooted neighbor joining tree (N-J) was constructed using MEGA X, keeping all the other parameters as default. To illustrate the exon/intron structure of all genes, the cDNA of all the genes were aligned with their genomic DNA using Gene Structure Display Server (GSDS) [21].

### 2.3. Functional Motifs and Domain Analysis

Conserved motifs were searched in gene sequences using online tool MEME suite [22], selecting the maximum number of motifs as 10, while all the other parameters were set as the default. All acquired motifs were arranged according to increasing E-value [23]. Using protein sequences, functional domains were searched by the SMART (Simple Modular Architectural Research Tool, http://smart.embl-heidelberg.de/, accessed on 7 August 2021) tool and drawn manually using GPS software [24].

### 2.4. Gene Location, Structural Prediction and 3D Modeling

After obtaining the location data of selected genes on chromosomes from the potato database Spud DB http://solanaceae.plantbiology.msu.edu/, accessed on 7 August 2021 [25], selected genes were spotted using the software, Phenogram (http://visualization.ritchielab.org/phenograms/plot, accessed on 7 August 2021). 

For 3D modeling, the online tool Swiss model was used (https://www.swissmodel.expasy.org/, accessed on 7 August 2021). Three genes, one from each group, were selected, which represent the models of all other highly similar genes. Modeling was done twice, firstly by using AtCWINV1 as a template, then a homologous model was predicted using the best-matched template using the template search option. All data were cross checked and retrieved using the protein database PDB (https://www.rcsb.org/ accessed on 7 August 2021) [26,27,28]. The 3D structures of *Solanum tuberosum* genes were firstly modeled using the Swiss model, keeping *AtCWINV1* as template.1 (Figure 8). Data were retrieved from the protein database (PDB ID: 2AC1), and the modeling was done by using the X-ray diffraction method with the resolution 2.15Å For more accuracy and to put light on the evolution, homologous modeling was done in addition, the template search was done again, and models were built using most homologous templates, Template.2 (Figure 8). The acquired models are represented according to the Q mean value; the blue color represents the high confidence portions, whereas the red color represents the low confidence regions.

### 2.5. Gene Expression Analysis in Different Tissues and Gene Ontology Annotation

RNA sequencing was done for leaves, petioles, above and below ground stolon, whole tuber, whole flower and flower parts, i.e., petals, sepals, stamens, carpels, mature and immature fruits, including a sample from the inside of fruits (mesocarp and endocarp), whereas the RNA sequencing of shoots and roots of 10–11-week-old callus from stem and leaves were taken from the in vitro propagated plants. To standardize the gene expression data from all samples, the FPKM (fragments per kilobase million) approach was adopted. After that, the retrieved data were normalized by using logarithmic base (log2) [29,30]. HemI (heat map illustrator) (http://hemi.biocuckoo.org/, accessed on 7 August 2021) was used to represent gene expression. The average hierarchical clustering method was used to plot the hierarchy on both X and Y axes, utilizing person distant similarity metrics [31]. The scale is represented by 15 distinct colors and values for expression.

Moreover, another heat map was plotted, representing the gene expression of invertase genes under biotic (*Phytophthora Infestans* for 24, 48, and 72 h) and abiotic stresses (Salt-150 mM NaCl, Mannitol-250 µM, BAP-10 µM, ABA 50 µM, IAA-10 µM, GA3-50 µM, Heat 35 °C for 24 h, primary and secondary tissue wounding, BABA and BTH treatment for 24 h/48 h/72 h).

Gene ontology enrichment analysis was firstly done by using the online GO enrichment tool PlantRegMap (http://plantregmap.cbi.pku.edu.cn/about.php/, accessed on 7 August 2021) by using gene IDs [32]. Primarily, this analysis was based on three main segments (cellular components, molecular functions and biological processes) but as a result of this analysis, no evident data about the functionality of invertase genes on the cellular component level were retrieved, so another analysis was done using software Blast2Go. Genomic sequences of invertase genes were uploaded. *Arabidopsis thaliana* was selected as the reference species for complete functional annotation.

### 2.6. Plant Materials Propagation Conditions and Treatments

- Growth conditions and Plant material

Initially, double monoploid plants (DM) were in vitro propagated (in 4.3 g/L MS salts, 0.8% agar, 3% sucrose, 0.17 g/L sodium phosphate, 2.5 mg/L thiamine, 0.1 g/L myo-inositol) with the 22 °C 16 h day light conditions, and 8 h 16 °C night conditions. Plants for control and heat 35 °C were maintained in the same conditions, whereas for salt, 150 mM NaCl and 260 µM mannitol plants were transferred to half-strength MS medium with 1.5% sucrose liquid medium after one week subjected to the abiotic stress for 24 h. For the collection of tubers, leaves, petioles and flowers, in vitro propagated plants were transferred to pots and maintained in the climate control growth chamber maintaining the same growth conditions. Flowers, leaves and petioles were collected and stored immediately at −80 °C.

B. qRT-PCR analysis

**Figure 1:**
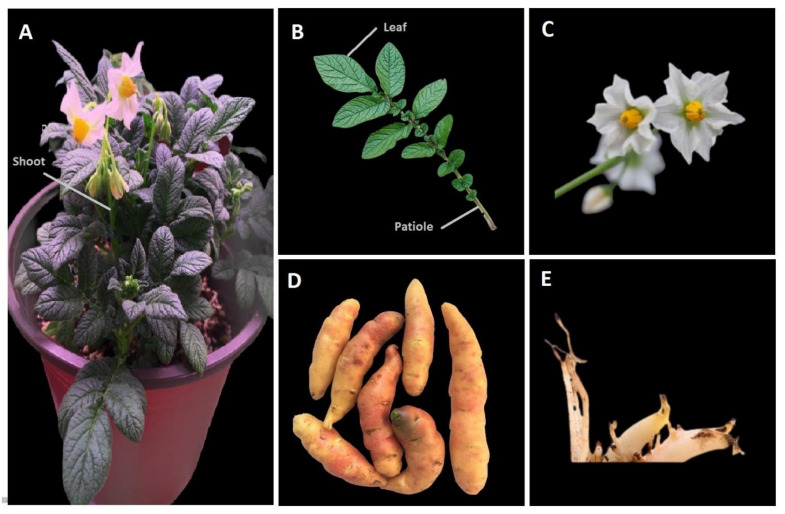
Potato plant tissues used for gene expression analysis. (**A**) Shoots, (**B**) patiole, (**C**) flower, (**D**) tuber, (**E**) roots.

The required tissue samples (Figure 1) were ground into powder in liquid nitrogen; 300~500 mg of the samples was used. Gene JET plant RNA extraction kit (Thermo Fisher Scientific, Yokohama, Japan) was used for RNA extraction. Afterwards, NanoDrop spectrometer (Thermo Fisher Scientific, Yokohama, Japan) was used for the quantification of RNA, and the extracted RNA was stored immediately at −80 °C to prevent degradation. PrimeScript™ along with gDNA Eraser (TaKaRa Bio, Kusatsu, Japan) was used for first strand cDNA synthesis from extracted RNA. All the reaction mixtures were prepared on ice following the manual instructions; synthesized cDNA was diluted to 100 ng, and immediately used for qRT-PCR analysis. Six of the *StINV* genes were selected for RNA sequence data gene validation depending upon their expression in different tissues. Relative gene expression was measured on IQ5 Realtime PCR system (BioRad, Hercules, CA, USA), using TB Green^®^ Premix Ex Taq™ II (Tli RNase H Plus, TaKaRa Bio, Japan) kit. Potato Actin97 was used as a reference gene to normalize the transcript level. The reaction mixture used was as follows: cDNA 0.5 μL, Forward Primer (10 µM) 0.25 μL, Reverse Primer (10 µM) 0.25 μL TB Green™ 5.0 μL, RNase free ddH_2_O 4.0 μL.

## 3. Results and Discussion

### 3.1. Genome Wide Analysis of Invertase Genes

**Figure 2:**
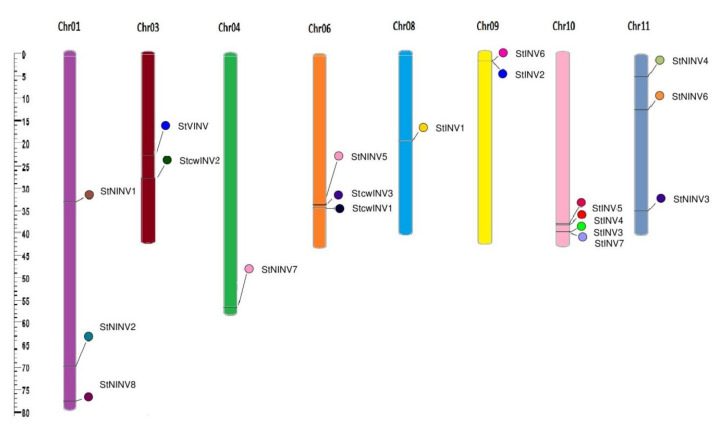
Locus map of invertase genes from *Solanum tuberosum*. Chromosomes are numbered and represented in different colors, whereas genes are represented at their positions.

A total of 21 genes were identified; two of them reflected incomplete protein sequences, so they were excluded. The remaining 19 identified genes in *Solanum tuberosum* were selected for further analysis, 11 of which are from sub family acidic invertases and 8 are from a neutral invertase sub family (shown in Table 1). Few of the genes have more than one transcript; in such cases, the primary transcripts were considered [33]. Genes were annotated manually and illustrated as *StVINV (Vacular Invertases)*, *StCWINV1–3 (Cell wall Invertases)*, *StINV1–7 (Invertases)*, and *StNINV1–8* [7]. Invertase family genes are unevenly distributed on chromosomes; the distribution varies from 4 to 0. Chromosome 10 had the highest number of invertase genes *StINV3/4/5/7*; chromosomes 1 and 11 had three neutral invertase genes each; chromosomes 3, 6, and 9 had two genes; the most functional genes were present on chromosome 3, i.e., (*StVINV* and *StCWINV2*); and the remaining chromosomes 4 and 8 just had one gene each (Figure 2). 

The protein length varies from 258 aa (*StCWINV3*) to 678 aa (*StNINV1*), whereas the size of most of the gene members ranges between 512aa and 655 aa. The information related to the gene id, transcript id, gene annotation, gene loci, gene orientation, chromosome location, protein length and accession numbers are given in (Table 1). We also identified invertase genes in *Arabidopsis thaliana* and *Solanum lycopersicum*, which were used for the phylogenetic analysis.

### 3.2. Phylogenetic Analysis and Gene Structure Illustration

**Figure 3:**
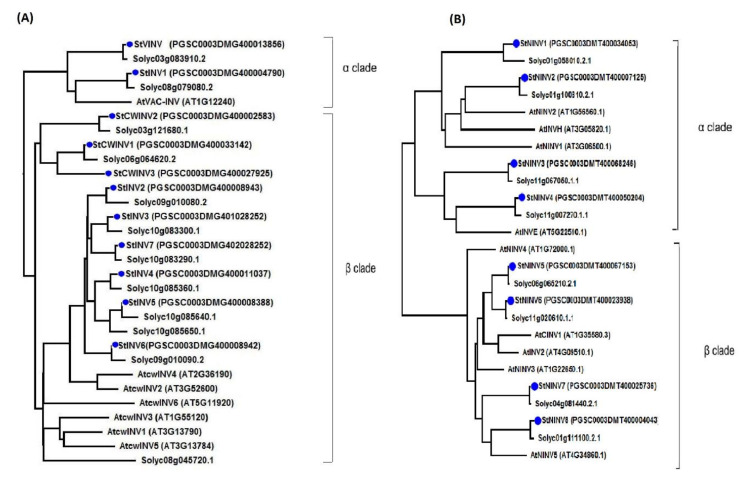
Phylogenetic tree of the acid invertase proteins and neutral/alkaline invertase proteins in *Solanum tuberosum*. (**A**) The α clade comprises vacuolar invertases, while the β clade is comprised of the cell wall and other members of invertase family. (**B**) The α clade is comprised of neural invertases (PtNINV1-4). The β clade is comprised of neutral invertases (PtNINV5-8).

**Figure 4:**
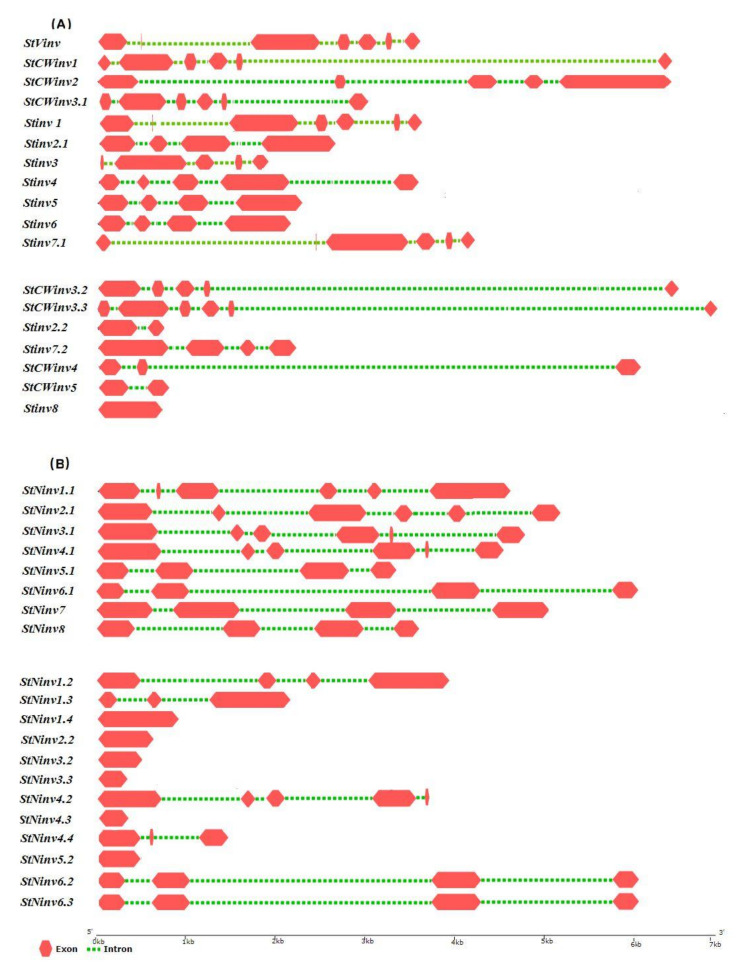
Symbolic structural representation of invertase gene family in *Solanum tuberosum*. Pink boxes represent exons and green dotted lines represent introns. (**A**) Acidic invertase sub-family. (**B**) Neutral/alkaline invertase sub-family.

For in depth understanding of the evolution and origin of gene homology, we performed multiple alignment using full-length protein sequences (Figures S2 and S3), constructed a phylogenetic tree (Figure 3) and illustrated the exons and introns of all the member genes (Figure 4). The invertase gene family was divided into two distinct sub classes, acid invertases and neutral invertases, represented by two phylogenetic trees (Figure 3). The phylogenetic analysis conceded that the acid invertase gene family can be segregated into α and β clades, where the α clade consists of vacuolar invertases consisting of *StVINV* and *StINV1*, whereas the β clade comprises cell wall invertase *StCWINV1–3* and other invertase members *StINV2–7* [34] (Figure 3A). Subfamily neutral/alkaline invertases also segregated into two clades α and β, where α consists of *StNINV 1–4* and the β clade consists of *StNINV 5–8* (Figure 3B). 

For in depth understanding of gene structure, genomic sequences and the corresponding cDNA sequence were submitted to the GSDS [21], which displays the genes structure. The number and size of the introns had a direct impact on the gene expression in response to the internal or external stimuli in plants [35]. *StVINV* has six introns, which is the maximum number of introns in the invertase gene family, which predicts its higher expression, whereas *StCWINV2* is intron-less (Figure 4A). Eight of the genes (42.1%) have three introns; 10.5% of the genes has four introns; and 38.6% of the genes has five introns (Figure 4). 

**Figure 5:**
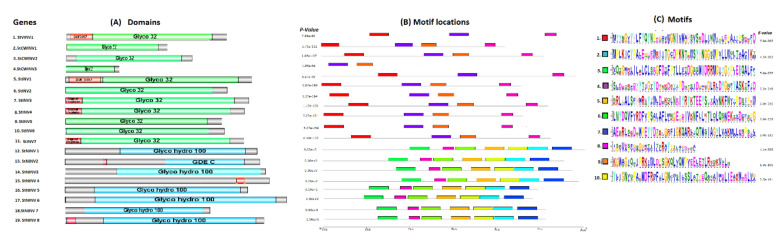
Graphical representation of functional domain and motifs. (**A**) Represents function domain on genes. (**B**) Shows different motifs located on the genes. (**C**) Motifs found by meme analysis.

Sequence comparison, in the acidic invertase sub family, explains that at the nucleotide level sequence, similarity ranges from 42.45% to 81.21% in the coding region, whereas in the amino acid level, it ranges from 36.76% to 81.21% (Table S1). In the case of alkaline/neutral invertases, the sequence identity at the nucleotide and amino acid levels ranges from 51.13% to 92.51% (Table S2). The conserved motifs were searched by MEME analysis; 10 motifs were found, and the details of the motifs found are represented in Figure 5C. Motifs 1, 2, 8 and 9 are highly conserved in acidic invertases, except in *StVINV* (motif 9 is absent), and *StCWINV3* just has motifs 2 and 9; on the other hand, in neutral/alkaline invertases, motifs 3–8 and 10 are highly conserved throughout in all genes (Figure 5B). 

### 3.3. Gene Structure Prediction and 3-Dimensional Modeling 

The sequence identity of the *StVINV* with Template.1 was 47.69% after modeling. The model was evaluated through the Q mean value, which was (−2.98). With Template.2 (6-fructosyl transferase), the sequence identity was 66.23% and the Q mean value was (−1.49) which is quite higher than in the previous Template.1. Structural analysis revealed the evolutionary relationship between the vacuolar invertases and fructosyltransferases. It provides the evidence that fructosyltransferases were evolved from vacuolar invertases [36]. The sequence similarity between the *StCWINV1* and Template.1 was 59.33% and the Q mean value was (−1.94). The sequence identity between *StCWINV1* and Template.2 (Beta-fructofuranosidase) was 59.13%. Modeling was done using X-ray diffraction with the resolution 2.80 Å. The two acquired models in the case of *StCWINV1* had the same attributes [2]. 

**Figure 6:**
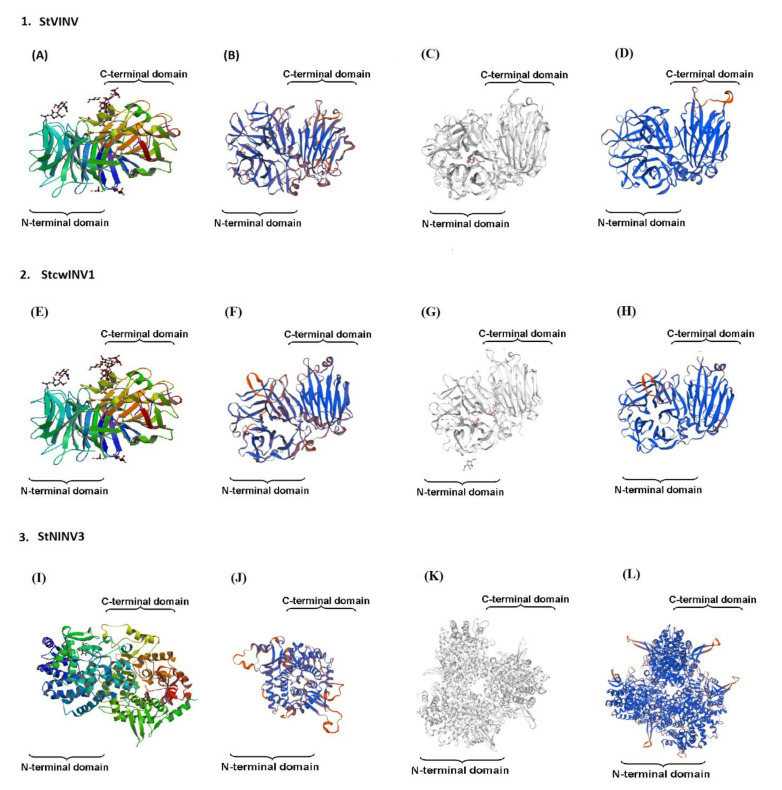
Predicted 3-dimensional structures of StVINV, StcwINV1, StNINV3. (**A**) AtCWINV1; (**B**) StVINV; (**C**) 6-fructosyltransferase; (**D**) StVINV; (**E**) AtCWINV1; (**F**) StcwINV1 (**G**) beta-fructofuranosidase; (**H**) StcwINV1; (**I**) AsInvB; (**J**) StNINV3 (**K**) AsInvA; (**L**) StNINV3. In the constructed 3D models bule color represents regions of high confidence and red color represents regions of low confidence.

For the 3-dimensional prediction of neutral/alkaline invertases, *StNINV3* alkaline/invertase gene InvB from *Anabaena* sp. PCC7021 (PDB ID; 5Z74) was selected as Template.1. Modelling was done by X-ray diffraction, using 1.95 Å resolution. It shared 58.35% sequence similarity, and the Q mean value was (−2.8). The Template.2 chosen was *InvA* from the same species. The sequence similarity shared was 55.88%, and the Q mean value was (−1.81). Structural comparisons revealed the evolutionary relation between the neutral/alkaline invertases of *Anabaena* sp. and higher plants (Figure 6) [37,38].

### 3.4. Gene Expression Pattern in Selected Tissues

**Figure 7:**
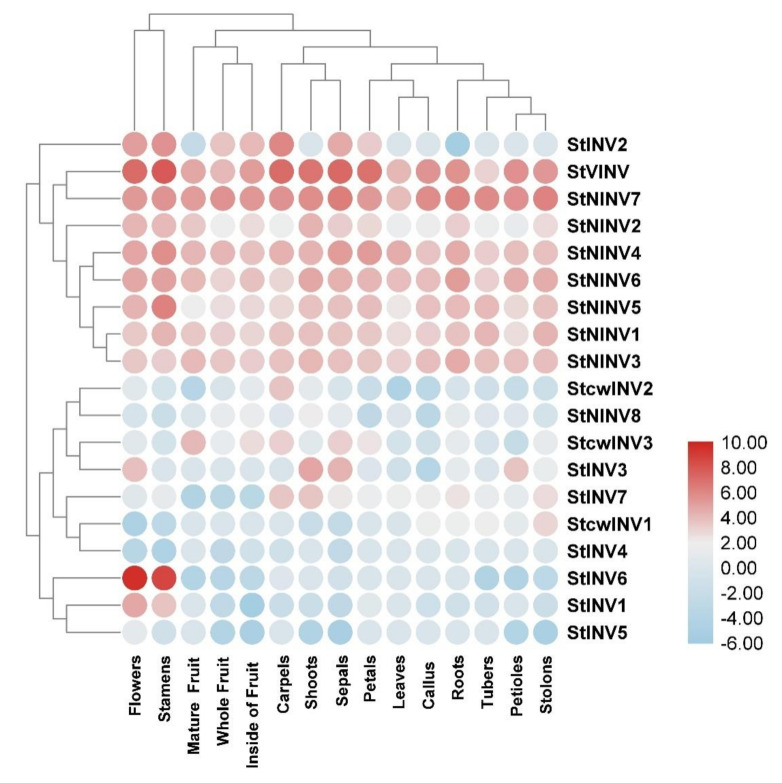
Heat map shows expression profile of invertase gene family in different tissues and organs of *Solanum tuberosum*. M, IM, IS are the short forms used for mature fruit, immature fruit and inside of fruit (mesocarp and endocarp).

For understanding the expression pattern of the invertase genes, the RNA-seq data of different tissues, such as leaves, petioles, shoots, stolon, roots, tubers, flowers, petals, sepals, stamens, carpels, callus, mature, immature and fruit (mesocarp and endocarp), were collected by fragments per kilobase of transcript per million mapped reads (FPKM) and used to plot the heat map. Among all genes, *StVINV* had the highest expression. It was expressed in almost all the tissues, but it had the highest expression in mature fruit, following tubers, flowers, stamens, carpels, and immature fruit, whereas comparatively low expression was noticed in callus leaves and stolon. These results are in accordance with the previous study [3]. However, in the cell wall invertases, *StCWINV1–2* showed very low expression in all tissues and they had no expression in the tuber, flower, petal, carpel, and mature fruit. *StCWINV3* was a bit expressive, as compared to the other cell wall invertases and had the highest expression in roots. In other acidic invertase genes, *StINV1* had expression in all the tissues, and *StINV2/3/4* showed similar expression patterns; they just had minor expression in the flower parts. *StINV5* showed low expression in the tuber, petiole and flower, whereas *StINV6* showed no expression (Figure 7) [39].

In the sub family, neutral/alkaline invertases *StNINV1* showed high expression in carpel and mature fruit, whereas it had low to no expression in other tissues. *StNINV2-6* showed an intermediate expression pattern between different tissues. For further in-depth understanding of the gene expression pattern, we designed a qRT-PCR analysis to quantify the expression patterns of selected genes. 

### 3.5. Gene Expression Analysis under Different Biotic and Abiotic Stresses 

Invertase genes are responsive to biotic and abiotic stresses; in order to check gene expression patterns under stress, the plants were subjected to biotic (*Phytophthora Infestans* for 24, 48, and 72 h) and abiotic stresses (Salt-150 mM NaCl, Mannitol-250 µM, BAP-10 µM, ABA 50 µM, IAA-10 µM, GA3-50 µM, heat 35 °C for 24 h, primary and secondary tissue wounding, BABA and BTH treatment for 24 h/48 h/72 h). Under salt and mannitol stress *StVINV*, *StCWINV1-3*, *StINV3* and *StNINV2-8* up-regulated significantly but *StINV1* and *StNINV1/5* were upregulated by salt treatment. Mannitol did not have a significant effect on the expression of these genes, whereas *StINV2/7* genes were negatively regulated by salt treatment. 

**Figure 8:**
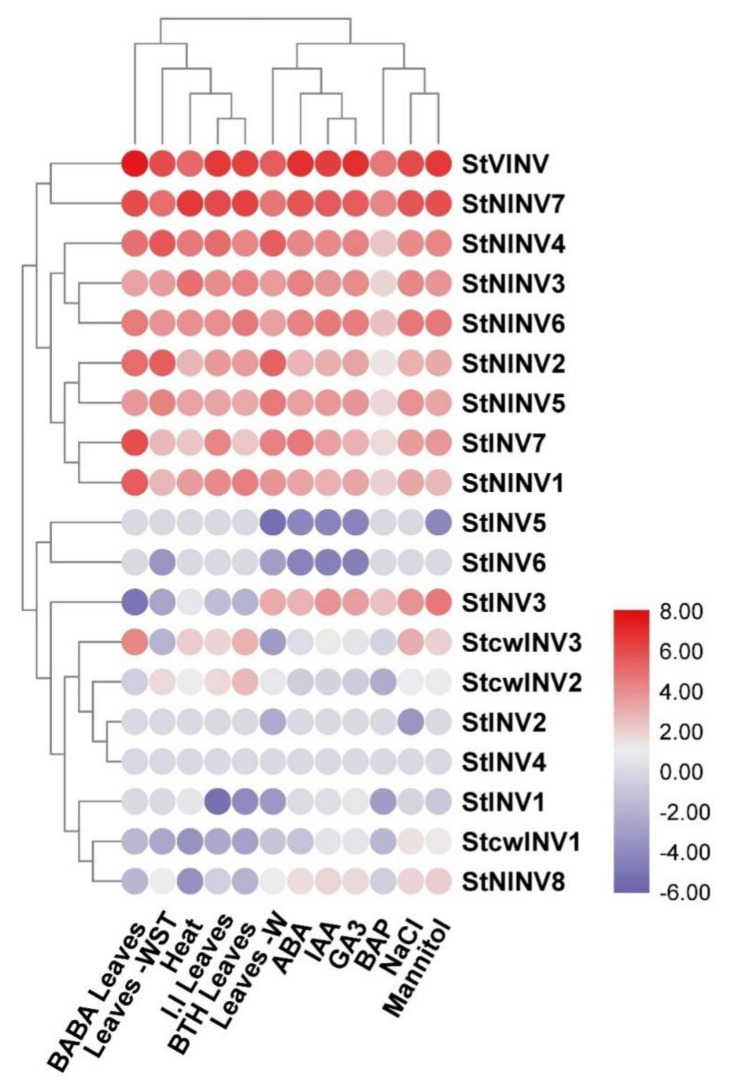
Heat map showing expression profile of invertase genes under different biotic and abiotic stresses in *Solanum tuberosum*. The short forms used are BAP: 6-benzylaminopurine6-benzylaminopurine, IAA: indole acetic acid, GA3: gibberellic acid, BABA: beta amino butyric acid, BTH: benzothiadiazole.

There was a down regulating trend in most of the genes under BAP, ABA, IAA, and GA3 treatments, except for a few genes: *StVINV* and *StNINV1/4* were up-regulated by the ABA and GA3 treatments; in addition, *StINV3* was upregulated by IAA treatment; and *StINV1* and *StNINV4* were upregulated by GA3 treatment. Under heat treatment, six of the genes were up-regulated, whereas all the other genes were down regulated. When primary and secondary tissue wounding was selected as treatment, all genes including *StVINV*, *StINV7* and *StNINV1-8* were highly expressed, while *StCWINV1-3* and *StINV1-6* showed low expression. Under BABA and BTH treatments, *StCWINV1-3* and *StINV1-7* showed low expression in comparison to other gene members. In response to the *Phytophthora Infestans*, the expression of most of the genes was down regulated, but in *StCwINV3*, *StINV3*, and *StNINV7*, a slight upregulating trend was evident (Figure 8). 

### 3.6. Validation of Invertase Gene Expression in Different Tissues

**Figure 9:**
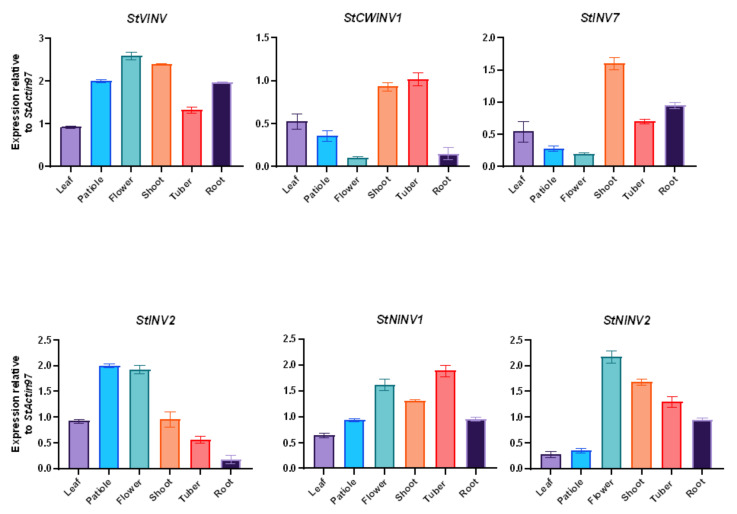
Expression pattern of invertase genes in different tissues. Fold change values are plotted, *StActin97* is used as internal control for three biological replicates of each sample in experiment. Bars are drawn for SD.

Primarily, different plant tissues (leaf, petiole, flower, shoot, tuber and roots) were selected to validate the expression of six selected invertase genes through qRT-PCR analysis. *StVINV* had expression in almost all of selected tissues with the highest expression in the flower, following the shoot and petiole [40]. *StCWINV1* had the highest expression in the tuber and shoot. *StINV7* and *StINV2* showed interesting patterns, where *StINV7* showed high expression in the shoot, whereas *StINV2* showed high expression in the petiole and flowers. Neutral invertases *StNINV1* and *StNINV2* had higher expression in the flower, shoot and tubers (Figure 9) [41]. 

### 3.7. Validation of Invertase Gene Expression under Abiotic Stress Conditions

**Figure 10:**
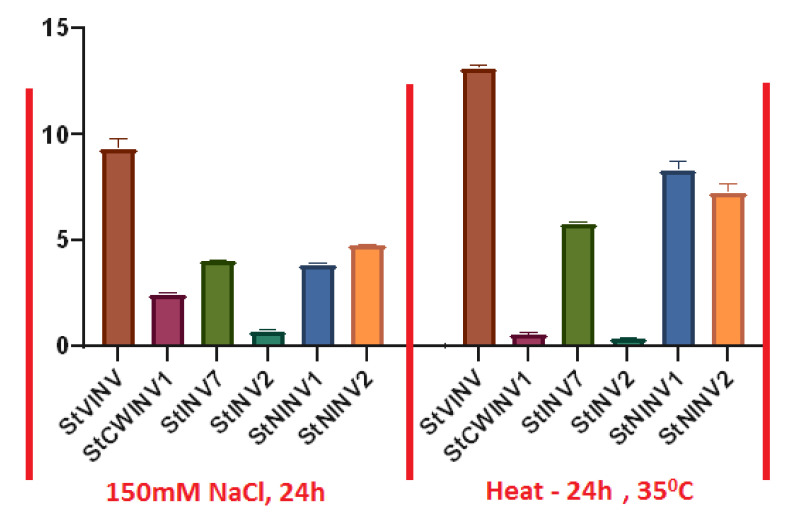
Expression pattern of invertase genes under salt and heat stress. Fold change values were plotted, *StActin97* was used as internal control for three biological replicates of each sample in the experiment. Bars are drawn for SD.

Gene expression of selected genes was firstly validated in the in vitro propagated plants under salt (150 mM NaCl, 24 h) and heat stress (35 °C, 24 h) by qRT-PCR (Figure 10). As a result of salt stress, *StVINV* was highly expressed followed by the neutral invertases. *StNINV1,2* and *StINV1* showed the lowest expression [40]. Due to heat stress *StVINV*, *StNINV1* and *StNINV2* had the highest expression. The gene expression pattern due to heat stress was similar to that of salt stress, but the overall expression was slightly higher than expected in *StINV2* and *StCWINV1*. These results are in accordance with [41,42]. 

**Figure 11:**
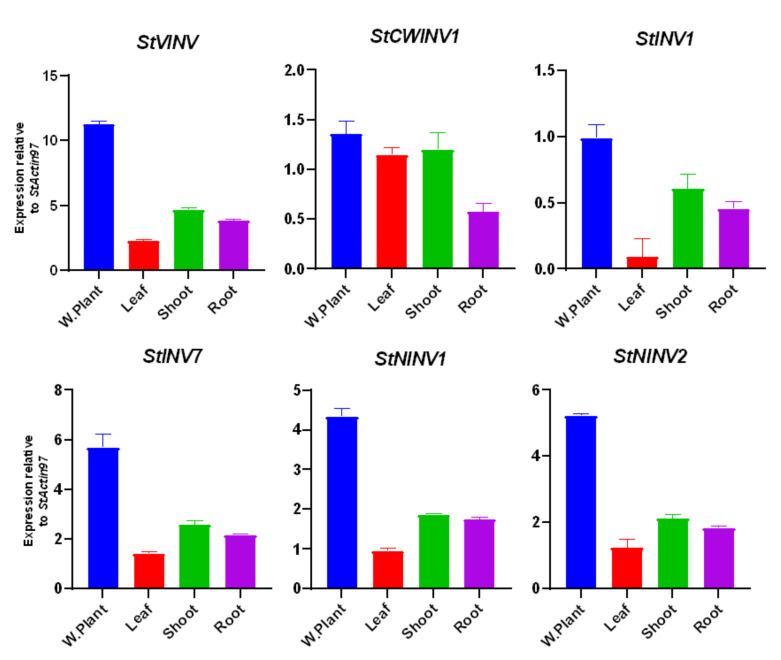
Expression pattern of invertase genes under drought stress. Fold change values are plotted, *StActin97* is used as an internal control for three biological replicates of each sample in the experiment. Bars are drawn for SD.

Along with salt and heat stress, we also validated gene expressions for drought stress, as with the changing environment, the world is now prone to drought stress more than before and very little is known about the roles and expression patterns of invertase genes during drought [43]. Owing to its importance along with the expression in the whole plant, we also performed qRT-PCR gene expression analysis additionally for leaf, root and shoot tissues (Figure 11). During drought *StVINV* and *StNINV1/2* are highly expressed in the whole plant [44,45]. When we compared other tissues, the shoots had the highest expression of all genes, followed by the roots. Low expression was noticed in leaves, probably due to the lower photosynthesis rate due to stress [46,47]. Low expression of *StCWINV* was noticed during drought. The stress results are consistent with [48,49,50]. 

### 3.8. GO Annotation of Solanum Tuberosum Invertase Proteins 

Gene ontology enrichment analysis was done in two steps firstly by using the online GO enrichment tool (PlantRegMap), using the gene n. of the invertase genes, which provides us with the genes’ involvement in molecular and biological processes. However, no evident data were present about their involvement in different processes at the cellular level. So, gene ontology enrichment analysis was performed again, using Blast2go software. The *Arabidopsis thaliana* results showed that in the molecular processes, genes were highly involved in hydrolase activity (hydrolyzing O-glycosyl compounds) and partially involved in the beta-fructofuranosidase activity, but showed very low catalytic activities. In biological processes, these genes are highly involved in sucrose and carbohydrate catabolic activities with very low involvement in the disaccharide catabolic processes and responses to wounding nectar secretion and responses to fungal infections. At the cellular level, the results showed that they are highly present in the apoplast and cell wall regions, with comparatively low occurrence in vacuole plasma membrane and integral components of the membrane (Figure S4). However, the transcriptional regulatory mechanism behind the endorsement of abiotic stress conditions is also important from the sustainability perspective [49,50].

## 4. Conclusions

This study identified 19 invertase genes in the *Solanum tuberosum* genome. Phylogenetic and sequence analyses revealed high similarity in their sequences, but functional divergence was noticed due to evolution. Expression analysis under different abiotic stress conditions revealed a highly diversified expression pattern in invertase family genes. For functional analysis, there is a need for further genetic and epigenetic studies.

## Figures and Tables

**Table 1:** Invertase family member genes.

GENE ID	NAMES	START	END	CHROMOSOME	ORIENTATION
PGSC0003DMG400013856	*StVINV*	39255144	39259094	3	Forward
PGSC0003DMG400002583	*StcwINV1*	61076945	61079609	6	Forward
PGSC0003DMG400027925	*StcwINV2*	27921873	27923870	3	Forward
PGSC0003DMG400033142	*StcwINV3*	47626455	47633517	6	Forward
PGSC0003DMG400004790	*StINV1*	52701644	52705608	8	Forward
PGSC0003DMG400008943	*StINV2*	2474975	2477132	9	Reverse
PGSC0003DMG401028252	*StINV3*	55840813	55843004	10	Forward
PGSC0003DMG400011037	*StINV4*	53706687	53710614	10	Reverse
PGSC0003DMG400008388	*StINV5*	53235938	53238048	10	Reverse
PGSC0003DMG400008942	*StINV6*	2469738	2471543	9	Reverse
PGSC0003DMG402028252	*StINV7*	55851974	55856628	10	Forward
PGSC0003DMG400013088	*StNINV1*	37067454	37072142	1	Reverse
PGSC0003DMG400002756	*StNINV2*	78430399	78435789	1	Reverse
PGSC0003DMG400026530	*StNINV3*	39908750	39913516	11	Forward
PGSC0003DMG400019494	*StNINV4*	5048165	5052692	11	Forward
PGSC0003DMG400026107	*StNINV5*	46662131	46665477	6	Forward
PGSC0003DMG400009257	*StNINV6*	13677099	13683141	11	Forward
PGSC0003DMG400009936	*StNINV7*	70804924	70808440	4	Reverse
PGSC0003DMG400001596	*StNINV8*	87160865	87164427	1	Reverse

## Data Availability

Data can be provided on suitable request.

## Supplementary Materials

The following are available online at https://www.mdpi.com/article/10.3390/biology11040539/s1, Figure S1: Equation showing 3-dimensional structures of sucrose, glucose, fructose and cleavage of sucrose in to glucose and fructose under the catalytic action of invertasetitle; Figure S2. Multiple alignment of sub-family Acid invertases in *Solanum tuberosum*. Fully conserved regions are represented by (*), Regions conserved between the groups which are highly similar in properties are represented by (:), (.) represents the conservation between groups having low similarities in properties.; Figure S3. Multiple alignment of sub-family Neutral/Alkaline invertases in *Solanum tuberosum*. Fully conserver regions are represented by (*), Regions conserved between the groups which are highly similar in properties are represented by (:), (.) represents the conservation between groups having low similarities in properties.; Figure S4. Gene ontology annotation of invertase proteins. (A) and (B) are showing gene annotation in potato. (B) (C) and (D) shows gene annotation using Arabidopsis thaliana as reference organism.; Table S1: Coding region nucleotide (upper portion of matrix) and amino acid (bottom portion of matrix) sequence pairwise comparison (% identity) between *Solanum tuberosum* acid invertase sub-family genes.; Table S2: Coding region nucleotide (upper portion of matrix) and amino acid (bottom portion of matrix) sequence pairwise comparison (% identity) between *Solanum tuberosum* Neutral/Alkaline invertase sub-family genes.

## References

[1] Roitsch T., Gonzalez M.C. (2004). Function and regulation of plant invertases: Sweet sensations. Trends Plant Sci..

[2] Lammens W., Le Roy K., Van Laere A., Rabijns A., Van den Ende W. (2008). Crystal structures of Arabidopsis thaliana cell-wall invertase mutants in complex with sucrose. J. Mol. Biol..

[3] Shen L.B., Qin Y.L., Qi Z.Q., Niu Y., Liu Z.J., Liu W.X., He H., Cao Z.M., Yang Y. (2018). Genome-Wide Analysis, Expression Profile, and Characterization of the Acid Invertase Gene Family in Pepper. Int. J. Mol. Sci..

[4] Ruan Y.-L., Jin Y., Yang Y.-J., Li G.-J., Boyer J.S. (2010). Sugar Input, Metabolism, and Signaling Mediated by Invertase: Roles in Development, Yield Potential, and Response to Drought and Heat. Mol. Plant.

[5] Draffehn A.M., Meller S., Li L., Gebhardt C. (2010). Natural diversity of potato (*Solanum tuberosum*) invertases. BMC Plant Biol..

[6] Zhu X., Richael C., Chamberlain P., Busse J.S., Bussan A.J., Jiang J., Bethke P.C. (2014). Vacuolar invertase gene silencing in potato (*Solanum tuberosum* L.) improves processing quality by decreasing the frequency of sugar-end defects. PLoS ONE.

[7] Chen Z., Gao K., Su X., Rao P., An X. (2015). Genome-Wide Identification of the Invertase Gene Family in Populus. PLoS ONE.

[8] Yao Y., Geng M.-T., Wu X.-H., Liu J., Li R.-M., Hu X.-W., Guo J.-C. (2014). Genome-wide identification, 3D modeling, expression and enzymatic activity analysis of cell wall invertase gene family from cassava (*Manihot esculenta* Crantz). Int. J. Mol. Sci..

[9] Sherson S.M., Alford H.L., Forbes S.M., Wallace G., Smith S.M. (2003). Roles of cell-wall invertases and monosaccharide transporters in the growth and development of Arabidopsis. J. Exp. Bot..

[10] Bhaskar P.B., Wu L., Busse J.S., Whitty B.R., Hamernik A.J., Jansky S.H., Buell C.R., Bethke P.C., Jiang J. (2010). Suppression of the vacuolar invertase gene prevents cold-induced sweetening in potato. Plant Physiol..

[11] Zhu X., Gong H., He Q., Zeng Z., Busse J.S., Jin W., Bethke P.C., Jiang J. (2016). Silencing of vacuolar invertase and asparagine synthetase genes and its impact on acrylamide formation of fried potato products. Plant Biotechnol. J..

[12] Brain P., Sagan D., Argulis L.M., Sagan D. (2008). Acquiring Genomes: A Theory of the Origin of Species.

[13] Ji X., Van den Ende W., Van Laere A., Cheng S., Bennett J. (2005). Structure, evolution, and expression of the two invertase gene families of rice. J. Mol. Evol..

[14] Vargas W., Cumino A., Salerno G.L. (2003). Cyanobacterial alkaline/neutral invertases. Origin of sucrose hydrolysis in the plant cytosol?. Planta.

[15] Dahal K., Li X.-Q., Tai H., Creelman A., Bizimungu B. (2019). Improving Potato Stress Tolerance and Tuber Yield Under a Climate Change Scenario—A Current Overview. Front. Plant Sci..

[16] Wiberley-Bradford A., Busse J., Jiang J., Bethke P. (2014). Sugar metabolism, chip color, invertase activity, and gene expression during long-term cold storage of potato (*Solanum tuberosum*) tubers from wild-type and vacuolar invertase silencing lines of Katahdin. BMC Res. Notes.

[17] Thalmann M., Santelia D. (2017). Starch as a determinant of plant fitness under abiotic stress. New Phytol..

[18] Kumar J., Das S., Teoh S.L. (2018). Dietary Acrylamide and the Risks of Developing Cancer: Facts to Ponder. Front. Nutr..

[19] Swarbreck D., Wilks C., Lamesch P., Berardini T.Z., Garcia-Hernandez M., Foerster H., Li D., Meyer T., Muller R., Ploetz L. (2008). The Arabidopsis Information Resource (TAIR): Gene structure and function annotation. Nucleic Acids Res..

[20] Goodstein D.M., Shu S., Howson R., Neupane R., Hayes R.D., Fazo J., Mitros T., Dirks W., Hellsten U., Putnam N. (2012). Phytozome: A comparative platform for green plant genomics. Nucleic Acids Res..

[21] Hu B., Jin J., Guo A.Y., Zhang H., Luo J., Gao G. (2015). GSDS 2.0: An upgraded gene feature visualization server. Bioinformatics.

[22] Bailey T.L., Boden M., Buske F.A., Frith M., Grant C.E., Clementi L., Ren J., Li W.W., Noble W.S. (2009). MEME SUITE: Tools for motif discovery and searching. Nucleic Acids Res..

[23] Kumar S., Stecher G., Li M., Knyaz C., Tamura K. (2018). MEGA X: Molecular Evolutionary Genetics Analysis across Computing Platforms. Mol. Biol. Evol..

[24] Liu W., Xie Y., Ma J., Luo X., Nie P., Zuo Z., Lahrmann U., Zhao Q., Zheng Y., Zhao Y. (2015). IBS: An illustrator for the presentation and visualization of biological sequences. Bioinformatics.

[25] Waterhouse A., Bertoni M., Bienert S., Studer G., Tauriello G., Gumienny R., Heer F.T., de Beer T.A.P., Rempfer C., Bordoli L. (2018). SWISS-MODEL: Homology modelling of protein structures and complexes. Nucleic Acids Res..

[26] Guex N., Peitsch M.C., Schwede T. (2009). Automated comparative protein structure modeling with SWISS-MODEL and Swiss-PdbViewer: A historical perspective. Electrophoresis.

[27] World Wide Web Consortium (2018). Protein Data Bank: The single global archive for 3D macromolecular structure data. Nucleic Acids Res..

[28] Schwede T., Kopp J., Guex N., Peitsch M.C. (2003). SWISS-MODEL: An automated protein homology-modeling server. Nucleic Acids Res..

[29] Filloux C., Cédric M., Romain P., Lionel F., Christophe K., Dominique R., Abderrahman M., Daniel P. (2014). An integrative method to normalize RNA-Seq data. BMC Bioinform..

[30] Deng W., Wang Y., Liu Z., Cheng H., Xue Y. (2014). HemI: A toolkit for illustrating heatmaps. PLoS ONE.

[31] Jin J., Tian F., Yang D.-C., Meng Y.-Q., Kong L., Luo J., Gao G. (2017). PlantTFDB 4.0: Toward a central hub for transcription factors and regulatory interactions in plants. Nucleic Acids Res..

[32] Park S., Shi A., Mou B. (2020). Genome-wide identification and expression analysis of the CBF/DREB1 gene family in lettuce. Sci. Rep..

[33] Juárez-Colunga S., López-González C., Morales-Elías N., Massange-Sánchez J., Trachsel S., Tiessen A. (2018). Genome-wide analysis of the invertase gene family from maize. Plant Mol. Biol..

[34] Ren X.Y., Vorst O., Fiers M.W., Stiekema W.J., Nap J.P. (2006). In plants, highly expressed genes are the least compact. Trends Genet. TIG.

[35] Lammens W., Le Roy K., Yuan S., Vergauwen R., Rabijns A., Van Laere A., Strelkov S.V., Van den Ende W. (2012). Crystal structure of 6-SST/6-SFT from Pachysandra terminalis, a plant fructan biosynthesizing enzyme in complex with its acceptor substrate 6-kestose. Plant J..

[36] Xie J., Hu H.X., Cai K., Xia L.Y., Yang F., Jiang Y.L., Chen Y., Zhou C.Z. (2018). Structural and enzymatic analyses of Anabaena heterocyst-specific alkaline invertase InvB. FEBS Lett..

[37] Xie J., Cai K., Hu H.X., Jiang Y.L., Yang F., Hu P.F., Cao D.D., Li W.F., Chen Y., Zhou C.Z. (2016). Structural Analysis of the Catalytic Mechanism and Substrate Specificity of Anabaena Alkaline Invertase InvA Reveals a Novel Glucosidase. J. Biol. Chem..

[38] Yuan H.-Z., Pang F.-H., Cai W.-J., Chen X.-D., Zhao M.-Z., Yu H.-M. (2021). Genome-wide analysis of the invertase genes in strawberry (Fragaria×ananassa). J. Integr. Agric..

[39] Eom S.H., Rim Y., Hyun T.K. (2019). Genome-wide identification and evolutionary analysis of neutral/alkaline invertases in Brassica rapa. Biotechnol. Biotechnol. Equip..

[40] Liu C., Xi H., Chen X., Zhao Y., Yao J., Si J., Zhang L. (2021). Genome-wide identification and expression pattern of alkaline/neutral invertase gene family in Dendrobium catenatum. Biotechnol. Biotechnol. Equip..

[41] Wang L., Zheng Y., Ding S., Zhang Q., Chen Y., Zhang J. (2017). Molecular cloning, structure, phylogeny and expression analysis of the invertase gene family in sugarcane. BMC Plant Biol..

[42] Shah A.N., Tanveer M., Abbas A., Fahad S., Baloch M.S., Ahmad M.I., Saud S., Song Y. (2021). Targeting salt stress coping mechanisms for stress tolerance in Brassica: A research perspective. Plant Physiol. Biochem..

[43] Ji X.M., Raveendran M., Oane R., Ismail A., Lafitte R., Bruskiewich R., Cheng S.H., Bennett J. (2005). Tissue-Specific Expression and Drought Responsiveness of Cell-Wall Invertase Genes of Rice at Flowering. Plant Mol. Biol..

[44] Zhu C., Yang K., Li G., Li Y., Gao Z. (2021). Identification and Expression Analyses of Invertase Genes in Moso Bamboo Reveal Their Potential Drought Stress Functions. Front Genet..

[45] Morales F., Ancín M., Fakhet D., González-Torralba J., Gámez A.L., Seminario A., Soba D., Ben Mariem S., Garriga M., Aranjuelo I. (2020). Photosynthetic Metabolism under Stressful Growth Conditions as a Bases for Crop Breeding and Yield Improvement. Plants.

[46] Deng F., Zeng F., Shen Q., Abbas A., Cheng J., Jiang W., Chen G., Shah A.N., Holford P., Tanveer M. (2022). Molecular evolution and functional modification of plant miRNAs with CRISPR. Trends Plant Sci..

[47] Nishanth M.J., Sheshadri S.A., Rathore S.S., Srinidhi S., Simon B. (2018). Expression analysis of Cell wall invertase under abiotic stress conditions influencing specialized metabolism in Catharanthus roseus. Sci. Rep..

[48] Albacete A., Cantero-Navarro E., Großkinsky D.K., Arias C.L., Balibrea M.E., Bru R., Fragner L., Ghanem M.E., González M.d.l.C., Hernández J.A. (2014). Ectopic overexpression of the cell wall invertase gene CIN1 leads to dehydration avoidance in tomato. J. Exp. Bot..

[49] Abbas A., Shah A.N., Tanveer M., Ahmed W., Shah A.A., Fiaz S., Waqas M.M., Ullah S. (2022). MiRNA fine tuning for crop improvement: Using advance computational models and biotechnological tools. Mol. Biol. Rep..

[50] Shah A.N., Tanveer M., Abbas A., Yildirim M., Shah A.A., Ahmad M.I., Wang Z., Sun W., Song Y. (2021). Combating Dual Challenges in Maize Under High Planting Density: Stem Lodging and Kernel Abortion. Front. Plant Sci..

