# Supplementary material for: Genome-Wide Analysis of Invertase Gene Family, and Expression Profiling under Abiotic Stress Conditions in Potato
Source: Biology (Basel). 2022 Mar 31;11(4):539. doi: 10.3390/biology11040539 (PMC9032393; doi:10.3390/biology11040539)
Supplement: Supplementary file 1 [file biology-11-00539-s001.zip › biology-1509072-supplementary.pdf]

Supplementary Material

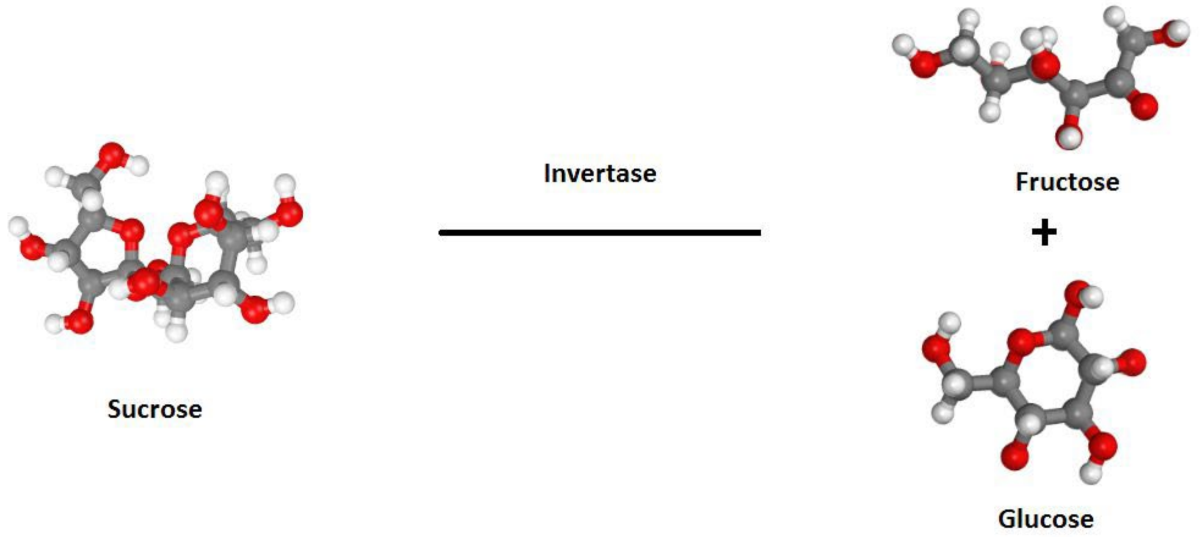

Figure S1. Equation showing 3-dimensional structures of sucrose, glucose, fructose and cleavage of sucrose in to glucose and fructose under the catalytic action of invertase

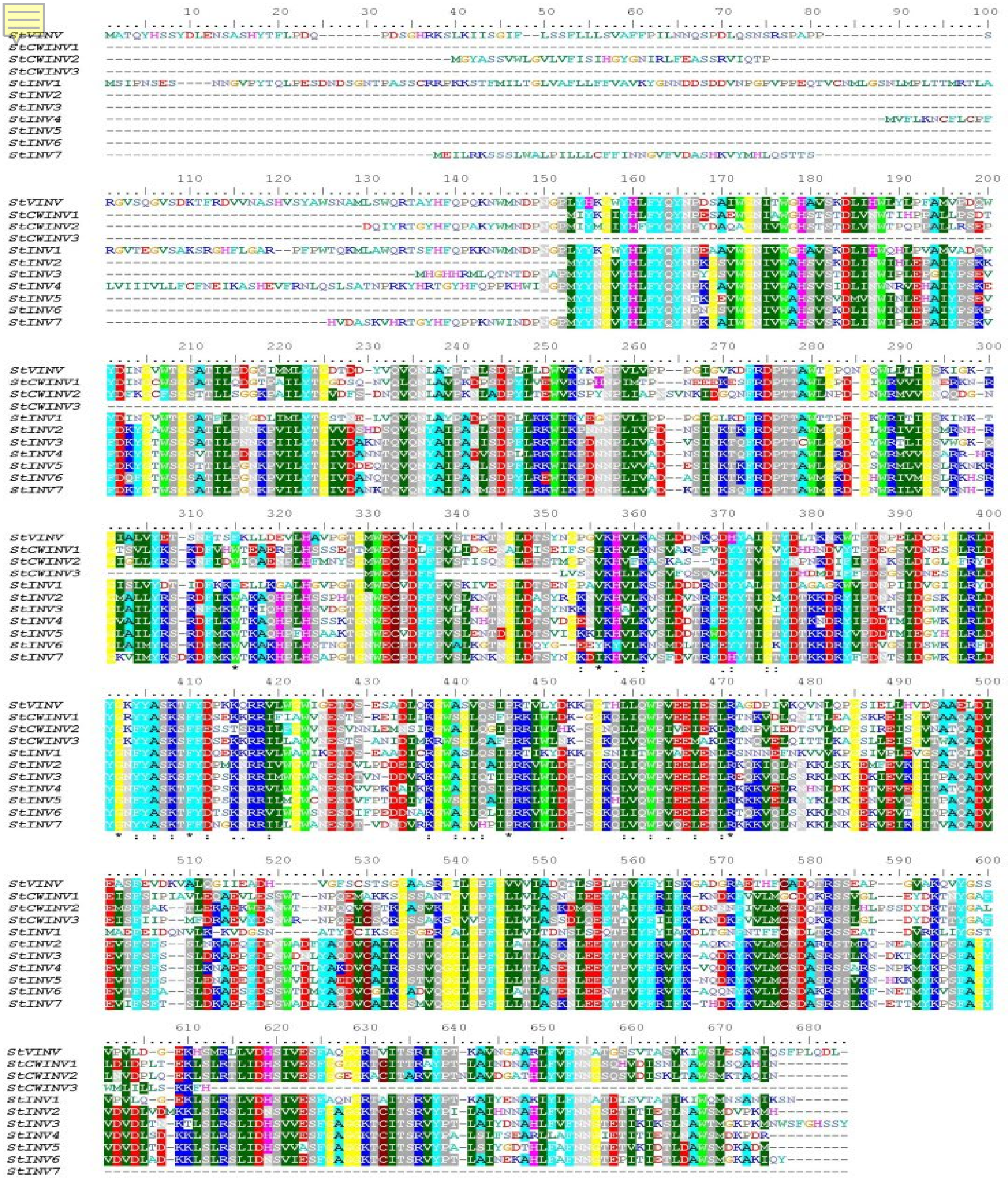

Figure S2. Multiple alignment of sub-family Acid invertases in *Solanum tuberosum*. Fully conserved regions are represented by (*), Regions conserved between the groups which are highly similar in properties are represented by (:), (.) represents the conservation between groups having low similarities in properties.

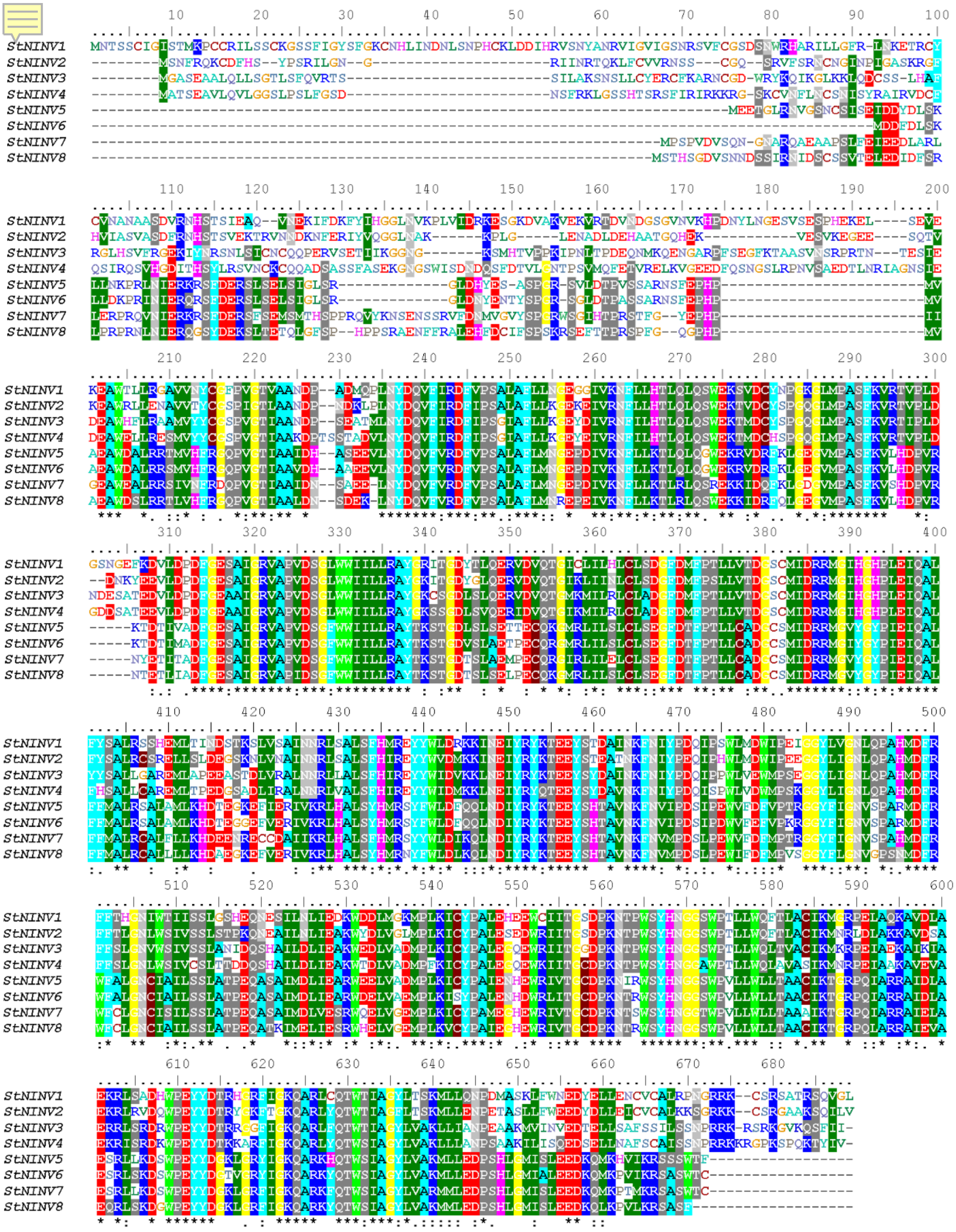

Figure S3. Multiple alignment of sub-family Neutral/Alkaline invertases in *Solanum tuberosum*. Fully conserver regions are represented by (*), Regions conserved between the groups which are highly similar in

properties are represented by (:), (.) represents the conservation between groups having low similarities in properties.

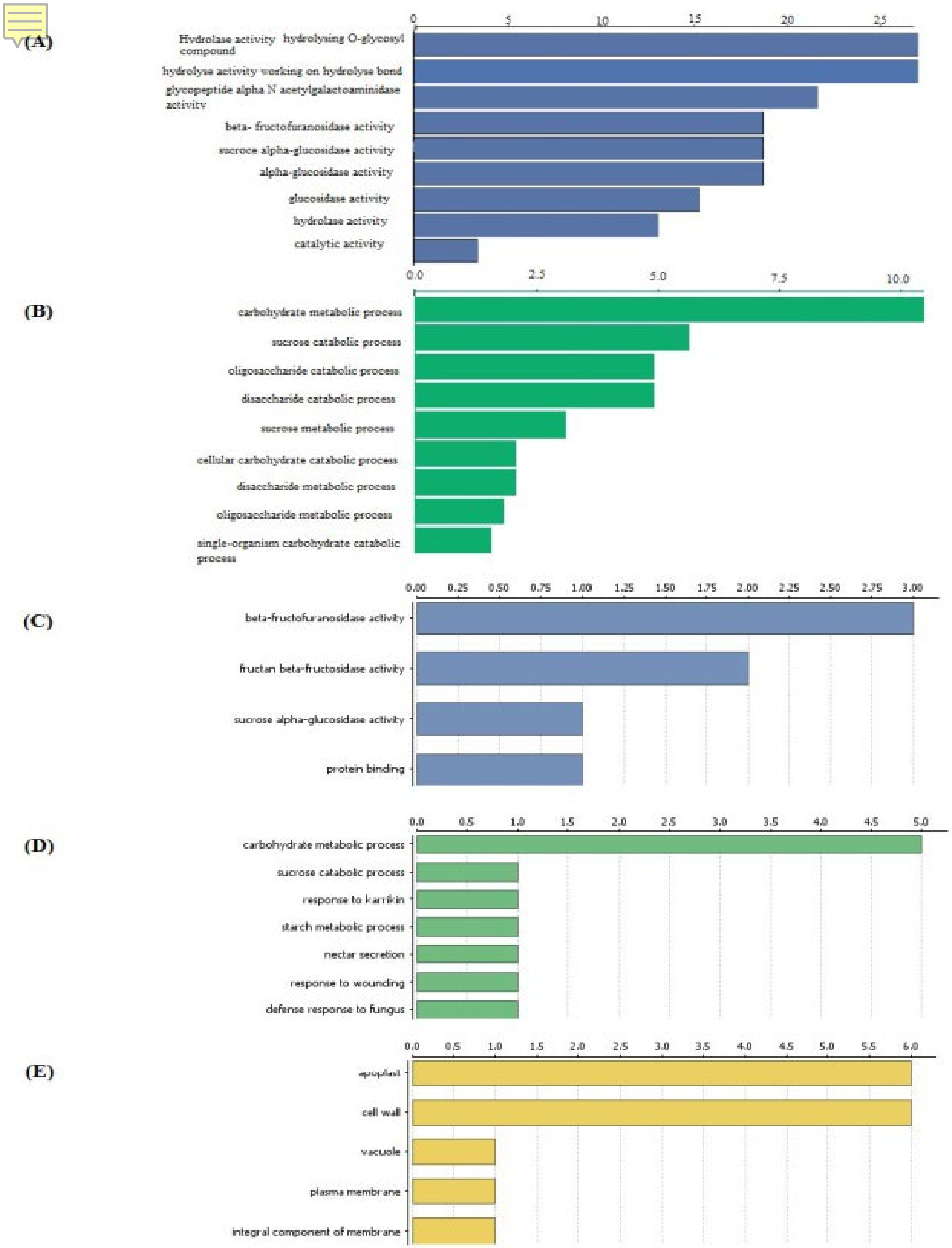

Figure S4. Gene ontology annotation of invertase proteins. (A) and (B) are showing gene annotation in potato. (B) (C) and (D) shows gene annotation using *Arabidopsis thaliana* as reference organism.

Table S1. Coding region nucleotide (upper portion of matrix) and amino acid (bottom portion of matrix) sequence pairwise comparison (% identity) between *Solanum tuberosum* acid invertase sub-family genes.

	StVINV	StINV1	StINV6	StINV4	StINV5	StINV7	StINV2	StINV3	StCWINV2	StCWINV1	StCWINV3 1:
StVINV	-	60.71	43.09	42.98	43.09	43.84	44.51	42.45	42.58	43.51	36.76
2: StINV1	60.71	-	43.78	43.27	43.37	44.42	45.40	42.55	42.50	44.60	38.10
3: StINV6	43.09	43.78	-	74.85	73.33	77.21	76.67	76.08	48.91	53.29	47.08
4: StINV4	42.98	43.27	74.85	-	81.21	75.66	78.08	76.53	47.24	53.78	50.97
5: StINV5	43.09	43.37	73.33	81.21	-	76.27	75.15	77.25	48.42	51.79	48.25
6: StINV7	43.84	44.42	77.21	75.66	76.27	-	77.30	78.98	49.91	55.18	52.34
7: StINV2	44.51	45.40	76.67	78.08	75.15	77.30	-	78.47	51.08	53.08	47.86
8: StINV3	42.45	42.55	76.08	76.53	77.25	78.98	78.47	-	49.14	54.58	51.17
9: StCWINV2	42.58	42.50	48.91	47.24	48.42	49.91	51.08	49.14	-	60.04	55.47
10:StCWINV1	43.51	44.60	53.29	53.78	51.79	55.18	53.08	54.58	60.04	-	68.99
11: StCWINV3	36.76	38.10	47.08	50.97	48.25	52.34	47.86	51.17	55.47	68.99	-

Table S2. Coding region nucleotide (upper portion of matrix) and amino acid (bottom portion of matrix) sequence pairwise comparison (% identity) between *Solanum tuberosum* Neutral/Alkaline invertase sub-family genes.

	StNINV8	StNINV7	StNINV5	StNINV6	StNINV3	StNINV4	StNINV1	StNINV2
1: StNINV8	-	76.28	78.90	78.83	51.79	51.13	51.73	52.93
2: StNINV7	76.28	-	78.43	79.66	51.88	51.88	51.91	52.45
3: StNINV5	78.90	78.43	-	92.51	53.54	52.96	53.52	52.10
4: StNINV6	78.83	79.66	92.51	-	54.94	53.29	54.37	54.04
5: StNINV3	51.79	51.88	53.54	54.94	-	69.26	58.12	62.24
6: StNINV4	51.13	51.88	52.96	53.29	69.26	-	57.41	61.09
7: StNINV1	51.73	51.91	53.52	54.37	58.12	57.41	-	70.25
8: StNINV2	52.93	52.45	52.10	54.04	62.24	61.09	70.25	-

S4 Primers Used

**CwINV1 F**	ATAGTATTAGAGGATGGGCTGGT
**CwINV1 R**	CCTGCGTAGCATTGACTCC
**CwINV3 F**	TAAAAATAGCGTGGCTCGTT
**CwINV3 R**	AAAAGACTTGGAAGCATAATACC
**INV1 F**	CGACCCATCGGACCCTC
**INV1 R**	AAAATCCACACATTCCCACATAC
**INV7 F**	CAAACACCCACTCCACTCAG
**INV7 R**	TTCCATCCATCAATAGAAGTGTT
**NINV1 F**	ATTTTGTCCCATCGGCTCT
**NINV1 R**	AGTCCCCTGTAATCCTTCC

**NINV2 F**	AAATCTGTGGTCCATTGTGTCA
**NINV2 R**	TGTGTTCTTGGGGTCGCTA
**VINV F**	CATCAAAGACATTTTATGACCCGAA
**VINV R**	TGTGTCCCTGTCTTCTTGTCGTAA
